# Response to “Evaluating Adverse Events in Databases”

**DOI:** 10.1002/prp2.1127

**Published:** 2023-08-17

**Authors:** Fabrizio Calapai, Carmen Mannucci, Luigi Cardia, Mariaconcetta Currò, Gioacchino Calapai, Emanuela Esposito, Ilaria Ammendolia

**Affiliations:** ^1^ Department of Clinical and Experimental Medicine University of Messina Messina Italy; ^2^ Department of Chemical, Biological, Pharmaceutical and Environmental Sciences University of Messina Messina Italy; ^3^ Department of Biomedical and Dental Sciences and Morphological and Functional Imaging University of Messina Messina Italy; ^4^ Department of Human Pathology of Adult and Childhood “Gaetano Barresi” University of Messina Messina Italy

Katie Lovell and Steven R. Feldman commented and criticized our recent manuscript entitled “Suspected oncologic adverse reactions associated with IL‐23 inhibitors in EudraVigilance: comparative study and gender distribution.” In the commentary, they list some methods to be used to determine the causal relationship between a drug and an adverse reaction. First of all, they cite randomized placebo‐controlled clinical trials, only to then consider them impracticable, due to the necessary time and resources. We think that other limitations, including small sample sizes and short study duration, could be easily added.^1^

An alternative method proposed is the use of a prospective registry. On this point, we agree and we think this research strategy is fundamental, since it provides high‐quality data from a specific patient population in a real‐world care setting. However, it is adopted in a more suitable way to verify a hypothesis, based on the suspicion that there is an association between an adverse event and the prescription of a drug. It is not the fastest way to discover new or rare adverse reactions.^2^ Another method evoked is the use of administrative claims data. Claims data might not be associated with detailed and accurate information because they are generally used for administrative or billing. Added to those cited by Lovell and Feldman, these limitations make still partial their usefulness.^3^

Spontaneous reporting database, the approach used by our group to evaluate the potential adverse reactions of IL‐23 inhibitors, is also debated by the commentary. The main function of this approach is the early identification of new, rare, and serious drug adverse reactions. It is generally recognized that lack of denominator, under‐reporting, Weber, and notoriety effect, prevent to draw definitive conclusions with this method.^4^ These problems are all cited and discussed in our manuscript. We also fully agree that methods to be associated with spontaneous reporting databases are needed and that they will improve the research activity in the field of pharmacovigilance.

Nevertheless, currently, we do not have the perfect foolproof method, since all the methods are presenting obstacles or limitations. Although, in this regard, among the various methods mentioned by Lovell and Feldman, only the obstacles of spontaneous reporting databases are criticized as “severe” limitations, even though, it is not possible to deny that their use is still the most common approach for the detection of adverse reactions.

The commentary asserted that we reach definitive conclusions based on data analysis of the database EudraVigilance. On the basis of the weakness points of all the methods, shared also in the commentary, it is possible to argue that at the moment in pharmacovigilance does not exist a methodological approach free of limitations to draw definitive conclusions. Therefore, also our conclusions cannot be considered as “definitive,” being accompanied by an examination of the limitations associated with the spontaneous reporting databases. Furthermore, also in the title of the manuscript, we defined as “suspected” the oncologic adverse reactions associated with IL‐23 inhibitors, asserting in this way the lack of certainty of the causal relationship between drug and event.

In the manuscript, we referred to the greater vulnerability of patients with psoriasis toward cancer. We called attention to the possibility, not the certainty, that there may be a link between the use of IL‐23 inhibitors and cancer. Furthermore, currently, there are no data excluding that IL‐23 inhibitors could or not potentiate this major susceptibility. As often happens in pharmacovigilance, this represents a signal that needs further studies producing in‐depth information, as close to the truth as possible, on the association between IL‐23 inhibitors and oncological adverse reactions. Development and acquisition of newer technologies to promote adverse reactions monitoring and reporting is a necessity for a more effective pharmacovigilance system.^5^ However, the shortcomings of spontaneous reporting are not a good reason not to accept the results of a method that until now served to discover important links between drugs and potential damage to health. So, even if results may be not pleasant, our duty is not to hide them behind comforting statements such as “in general, IL‐23 inhibitors are safe drugs typically well tolerated by patients.” Especially if in support of this statement, the commentary of Lovell and Feldman cites scientific articles published years ago that certainly do not discuss the use of IL‐23 inhibitors in the treatment of psoriasis.

In particular, the commentary cites the work of Fiorentino et al.,^6^ published in November 2017 (we analyzed spontaneous reports of adverse reactions for the years 2020–2022). This article does not deal with this topic and could not be differently because European authorizations of the IL‐23 inhibitors tildrakizumab (September 2018), risankizumab (April 2019), and guselkumab (November 2017), are all subsequent or contemporary^6^ to its publication.

Finally, we conclude this response reiterating that, despite known limitations, spontaneous reporting of adverse reactions is the cornerstone of pharmacovigilance that still today helps us to face address safety concerns after drug authorization. Despite its shortcomings, this inexpensive and effective method of collecting information keeps to inspire researchers by providing them the possibility to conduct independent research in pharmacovigilance. We thank Katie Lovell and Steven R. Feldman and the Journal for giving us the opportunity to clarify some aspects related to our research activity.

## AUTHOR CONTRIBUTIONS

All the authors gave substantial contributions to the conception of this response; they have been involved in writing and gave final approval of this version to be published.

## CONFLICT OF INTEREST STATEMENT

The authors have no conflicts of interest to disclose.

## CONSENT

Not applicable.

## ETHICS STATEMENT

Not applicable.

## Data Availability

Not applicable.
